# miR-139-5p Was Identified as Biomarker of Different Molecular Subtypes of Breast Carcinoma

**DOI:** 10.3389/fonc.2022.857714

**Published:** 2022-03-31

**Authors:** Haohang Sun, Ji Dai, Mengze Chen, Qi Chen, Qiong Xie, Weijun Zhang, Guoqing Li, Meidi Yan

**Affiliations:** ^1^ General Surgery I (Thyroid, Breast, Vascular, Hernia Surgery), General Hospital of Zhenhai District People’s Hospital Medical Group, Ningbo, China; ^2^ Department of General Surgery, Zhenhai District People’s Hospital, Ningbo, China

**Keywords:** miR-139-5p, mesenchymal stem cells, exosomes, molecular isoforms, breast carcinoma

## Abstract

Located on chromosome 11q13.4, miR-139-5p has been confirmed by several studies as a possible attractive biomarker for cancer, including breast cancer, but its mechanism of correlation in different molecular subtypes of breast cancer has not been reported. In this study, comprehensive bioinformatics analysis was used to evaluate the expression of miR-139-5p in different molecular subtypes of breast cancer (luminal A, luminal B, HER2-enriched, and basal-like). The target genes of miR-139-5p were predicted by using an online database TargetScan and miRDB, and three key genes, FBN2, MEX3A, and TPD52, were screened in combination with differentially expressed genes in different molecular subtypes of breast cancer. The expression of the three genes was verified separately, and the genes were analyzed for pathway and functional enrichment. Bone marrow mesenchymal stem cells (BMSC) are another kind of highly plastic cell population existing in bone marrow besides hematopoietic stem cells. BMSC can affect the proliferation and migration of cancer cells, promote the metastasis and development of cancer, and regulate the tumor microenvironment by secreting exosome mirnas, thus affecting the malignant biological behavior of tumor cells. Finally, human bone marrow mesenchymal stem cells exosomes were obtained by ultracentrifugation, and the morphology of exosomes was observed by transmission electron microscopy. The expression of miR-139-5p in normal breast cells MCF-10A, human breast cancer cell line MDA-MB-231 cells, and BMSCs-derived exosomes were compared; the exosomes and MDA-MB-231 cells were co-cultured to observe their effects on the proliferation of the MDA-MB-231 cells. Human bone marrow mesenchymal stem cell-derived exosomes inhibited the growth of breast cancer cells and promoted the expression of FBN2, MEX3A, and TPD52 by transporting miR-139-5p.

## Introduction

Breast cancer is a global female health problem (1). According to recent Global Cancer Observatory (GLOBOCAN) 2020 data, the incidence of breast cancer in women has surpassed lung cancer as the leading cause of cancer incidence worldwide, with an estimated 2.3 million new cases, accounting for 11.7% of all cancer cases (2). In addition, the incidence of breast cancer is rising rapidly in South America, Africa, and Asian countries in transition, as well as in high-income Asian countries (Japan, China, and Korea) (3, 4). Breast cancer is a complex heterogeneous tumor that can be classified according to histological features as hormone receptor (ER+, PR+/-), human epidermal growth factor receptor 2+ (HER2+), and triple-negative breast cancer (TNBC), also known as basal cell type, which are key factors affecting patient prognosis (5, 6). Although breast cancer treatment and early diagnosis have continued to improve and the 5-year survival rate of breast cancer patients has increased, breast cancer is still considered the second leading cause of cancer-related death (7).

Pathology plays a key role in understanding complex diseases such as cancer. However, in our country, data on key epidemiological findings are lacking, and studies have been conducted to show that the proportion of breast cancer subtypes varies in different populations (8). However, these traditional classifications do not reflect the diversity of breast cancers. For example, patients with HER2-negative or ER-negative tumors do not respond to neoadjuvant therapy targeting HER2. Women who are HER2-positive or ER-positive tend to show recurrent responses to such targeted therapy (9). To better predict patient prognosis, the overall classification of breast cancer has changed in the last decade or so. Microarray-based gene expression profiling helps to identify histopathological types and molecular subtypes of breast cancer (10). The Cancer Genome Atlas (TCGA), extensive analysis of protein levels, microRNA, and DNA based on the detection of ER, PR, HER2, and low expression proliferating cell nuclear antigen-67 (Ki-67) indicators have helped to establish a more refined subtype of breast cancer. Molecular subtypes include luminal A, luminal B, HER-enriched, and basal-like, each of which has changed the way breast cancer is treated (11). Each subtype is associated with a different incidence, prognosis, preferential metastatic organs, treatment response, recurrence, or disease-free survival outcomes (12–14).

MicroRNAs are single-stranded short non-coding RNAs, and their genes are usually found in clusters, distributed on all chromosomes except the Y chromosome (15). An important regulator, miR-139-5p is closely associated with the proliferation, invasion, and metastasis of a variety of tumors (16). Its mechanism of action in breast cancer has also been discovered by many studies. However, its mechanism of action in different molecular subtypes of breast cancer has not been overly reported. Besides, miRNAs loaded in exosomes have also been reported, demonstrating that exosomes may act as a miRNA transport to regulate intercellular communication (17) and exosomes secreted by the BMSCs have been reported to have therapeutic potential (18). Exosomes are small intraluminal vesicles that are secreted by various cells and can deliver intracellular contents, such as microRNAs (miRNAs), messenger RNAs (mRNAs) and proteins (19, 20). It is known that multiple target genes can exist simultaneously in the same miRNA (21). Therefore, this study investigated the expression of miR-139-5p and its target genes in different molecular subtypes of breast cancer, aiming to provide potential targets for the treatment of different molecular subtypes of breast cancer.

## Methods

### Data Sources

The RNA-Seq, miRNA-seq, and clinical information of patients with breast invasive carcinoma (BRCA) were downloaded from The Cancer Genome Atlas (TCGA) database (https://portal.gdc.cancer.gov/). If multiple probes were detecting the same miRNA expression during the analysis, the average of the miRNA expression was taken as the expression value of that miRNA. For the analysis of patient clinical information, the clinical information of patients with unknown survival time and survival time of 0 was deleted.

### Variance Analysis

Differentially expressed genes were screened using the edgeR package in R v4.0.3, with a |logFC| ≧ 1 and an adjust *P* value < 0.05. Volcano plots of differentially expressed genes were plotted using ggplot2.

### Kaplan-Meier Survival Analysis

The survival analysis was performed using Survival in the R package. The *p*-values and hazard ratios (HR) with 95% confidence intervals (CI) in the Kaplan-Meier curves were derived by log-rank test and univariate Cox proportional hazards regression.

### Gene Ontology and Kyoto Encyclopedia of Genes and Genomes Enrichment Analysis

The GO analysis and KEGG analysis of genes were performed using the DAVID 6.8 database (https://david.ncifcrf.gov/). Enrichment results with *p* < 0.05 or false discovery rate (FDR) < 0.05 were statistically significant.

### Gene Set Enrichment Analysis

The samples were divided into high and low expression groups according to the median gene expression, and RNA-seq profiles were loaded to GSEA to investigate key gene-related signaling pathways in the high and low-risk groups. FDR < 0.25, *P* < 0.05 were considered to be significantly enriched.

### Cell Source and Culture

The human normal breast cells MCF-10A, human breast cancer cell line MDA-MB-231 cells, and human bone marrow mesenchymal stem cells (BMSCs) were purchased from the American Type Culture Collection (ATCC) cell bank (Manassas, VA, USA). The MCF-10A cells were cultured in DMEM/F12 medium (Gibco) containing 2.5 mM glutamine, 20 ng/ml epidermal growth factor, 0.01 mg/ml insulin, 500 ng/ml hydrocortisone, and 5% horse serum in DMEM/F12 medium (Gibco). The MDA-MB-231 cells were cultured in RPMI 1640 medium (Gibco) containing 10% fetal bovine serum (FBS) and 0.5% penicillin-streptomycin. The BMSCs were cultured in DMEM medium (Gibco) containing 10% fetal bovine serum (FBS) and 1% penicillin-streptomycin in DMEM medium (Gibco). All cells were routinely cultured at 37°C in a 5% CO_2_ incubator.

### Isolation and Identification of Exosomes

The BMSCs were grown and fused to 80%, rinsed in phosphate buffered saline (PBS), and cultured in DMEM medium without exosomal fetal bovine serum for 2 d. The exosomes were extracted by ultracentrifugation: the cell culture medium was collected, centrifuged at 500r/min for 12 min to remove the cells, and then centrifuged at 5000r/min for 12 min to remove the cell fragments. Collect supernatant filtered by 0.22 μM membrane, low temperature ultracentrifugation for 2 h, take the precipitation and dissolve it in PBS for subsequent experiments. The morphology of exosomes was observed by transmission electron microscope. The exosome specific protein markers CD63, CD9 and HSP70 were detected by western blot.

### Cell Co-Culture with Exosome

The MDA-MB-231 cells were divided into a control and an exosome group. The cells were inoculated in 6-well plates 24 h before treatment, and when the growth fusion reached 70%, 200 μL of exosomes were added to each well of the exosome group and co-cultured with the cells for 48 h. and PBS was added to the control group, and the cells were collected for subsequent experiments.

### Quantitative Real-Time PCR

The total RNA was extracted from the cells using Trizol reagent (Invitrogen), and NanoDrop (Thermo Fisher Scientific) was applied to detect the concentration and purity of the RNA. The cDNA was reverse transcribed into cDNA according to the instructions of the PrimeScript RT Reagent Kit (Takara), and this cDNA was used as a template for PCR reactions, using the ABI Step One Real-time PCR System (Thermo Fisher Scientific) according to the instructions of the SYBR GREEN kit (TaKaRa). The specific primers used for qRT-PCR were synthesized by Shanghai Bioengineering Co (**Table 1**). The mean values were taken.

**Table 1:** Primer sequences.

Gene	Primer sequence (5’-3’)
miR-139-5p	Forward: CGCGTCTACAGTGCACGTGTC
	Reverse: AGTGCAGGGTCCGAGGTATT
FBN2	Forward: TGGATTTTGTTCCCGTCCTAAC
	Reverse: CAACGTCCACCATTCTGACAT
MEX3A	Forward: TGGAGAACTAGGATGTTTCGGG
	Reverse: GAGGCAGAGTTGATCGAGAGC
TPD52	Forward: AGCATCTAGCAGAGATCAAGCG
	Reverse: AGCCAACAGACGAAAAAGCAG
U6	Forward: CTCGCTTCGGCAGCACA
	Reverse: AACGCTTCACGAATTTGCGT
GAPDH	Forward: CTGGGCTACACTGAGCACC
	Reverse: AAGTGGTCGTTGAGGGCAATG

### CCK-8 Assay for Cell Proliferation Viability

The MDA-MB-231 cells in the logarithmic growth phase were inoculated with 1×10^4^ cells/well in a 96-well plate and incubated at 37°C for 24, 48, 72, and 96 h. Next, 10 μL of CCK-8 solution was added to each well and incubated continuously for 4 d at 37°C in a constant temperature incubator. Finally, the absorbance value of each well was measured at 450 nn, using an enzyme marker, and the absorbance value was used to represent the cell proliferation level.

### Statistical Methods

Statistical software (SPSS 21.0, IBM Corp. Armonk, USA) was used for data analysis, and Prism 8.0 (Graphpad, USA) was used for graphical presentation. Measures were expressed as mean standard deviation (Mean ± SD), and each experiment was repeated at least three times. Comparisons between two groups for measures that obeyed a normal distribution were performed using the independent sample t-test. Comparisons between multiple groups were made using a one-way ANOVA followed by Tukey’s *post hoc* test. A two-tailed *P* < 0.05 was considered statistically significant.

## Results

### Expression of miR-139-5p in Breast Cancer and Prognosis

**Figure 1:**
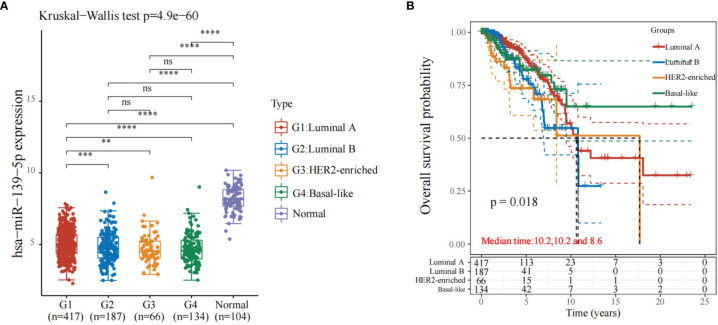
Expression and prognosis of miR-139-5p in different types of breast cancer. **(A)** Expression of miR-139-5p; **(B)** Prognosis of different types of breast cancer. **P < 0.01, ***P < 0.001, ****P < 0.0001. ns, no significance.

The expression of miR-139-5p in luminal A, luminal B, HER2-enriched, and basal-like breast cancers in the TCGA database was observed; the results showed that compared with normal samples, miR-139-5p expression was reduced in luminal A, luminal B, HER2-enriched, and basal-like breast cancers (**Figure 1A**). The differences pf the expression level of miR-139-5p between basal-like breast cancer and HER2-enriched breast cancer were statistically significant (P < 0.05). The prognosis of the four different types of breast carcinoma (**Figure 1B**) showed that there was a difference in prognosis between the four types of breast carcinoma (P < 0.05).

### Screening of Differentially Expressed Genes in Different Types of Breast Cancer

**Figure 2:**
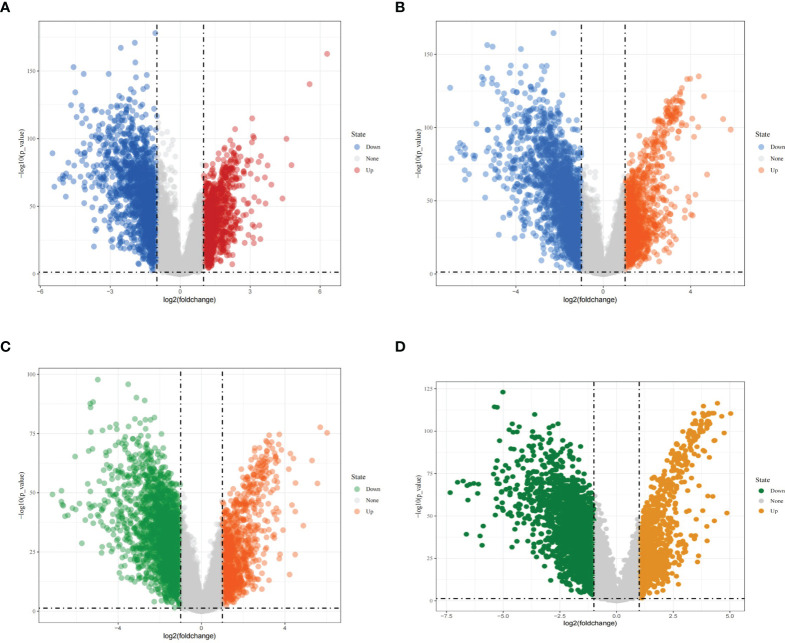
Differentially expressed genes in different types of breast cancer. **(A)** Volcano diagram showing differentially expressed genes in luminal A breast cancer; **(B)** Volcano diagram showing differentially expressed genes in luminal B breast cancer; **(C)** Volcano diagram showing differentially expressed genes in HER2-enriched breast cancer; **(D)** Volcano diagram showing differentially expressed genes in basal-like breast cancer.

Using |logFC|>1 and adjusted *P* < 0.05 as screening conditions, differentially expressed genes were screened from different types of breast cancer in the TCGA database and visualized, using volcano plots. A total of 1674 downregulated genes and 905 upregulated genes were obtained from the screening of luminal A breast cancer (**Figure 2A**); from the screening of luminal B breast cancer, 2270 downregulated genes and 1186 upregulated genes were obtained (**Figure 2B**); and from the screening of HER2-enriched breast cancer, 2513 downregulated genes and 974 upregulated genes were obtained (**Figure 2C**). A total of 2445 downregulated genes and 1092 upregulated genes (**Figure 2D**) were obtained in the basal-like breast cancer screen.

### Functional Enrichment Analysis of Differentially Expressed Genes in Four Types of Breast Cancer

**Figure 3:**
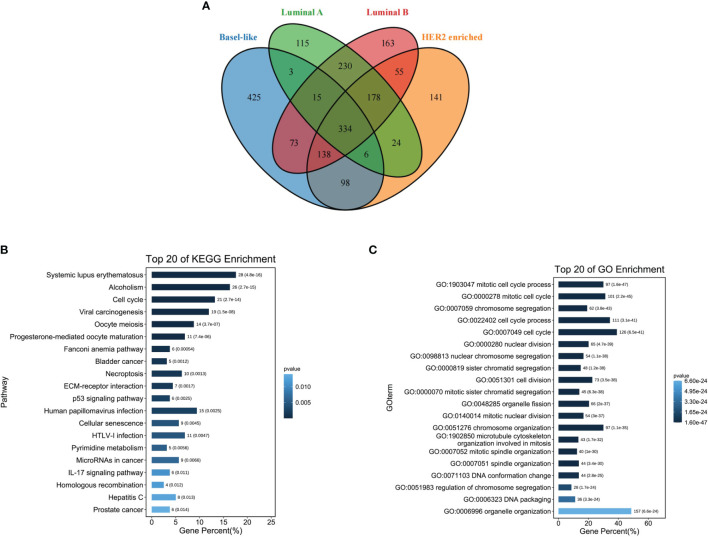
Functional enrichment analysis of differentially expressed genes in four types of breast cancer. **(A)** Venn diagram showing four types of breast cancer differentially up-regulated overlapping genes; **(B)** Overlapping gene KEGG pathway enrichment analysis; **(C)** Overlapping gene GO function enrichment analysis.

The differentially up-regulated genes in the four types of breast cancer overlapped, and a total of 334 overlapping genes were obtained (**Figure 3A**). Subsequently, the KEGG pathway analysis was performed on the 334 differentially up-regulated genes, which involved a total of 20 pathways, mainly enriched in the systemic lupus erythematosus pathway, alcohol moderate, cell cycle, and other pathways (**Figure 3B**). The GO enrichment analysis showed that 334 differentially upregulated genes were mainly enriched in the cell cycle (**Figure 3C**).

### Target Gene Prediction of miR-139-5p

**Figure 4:**
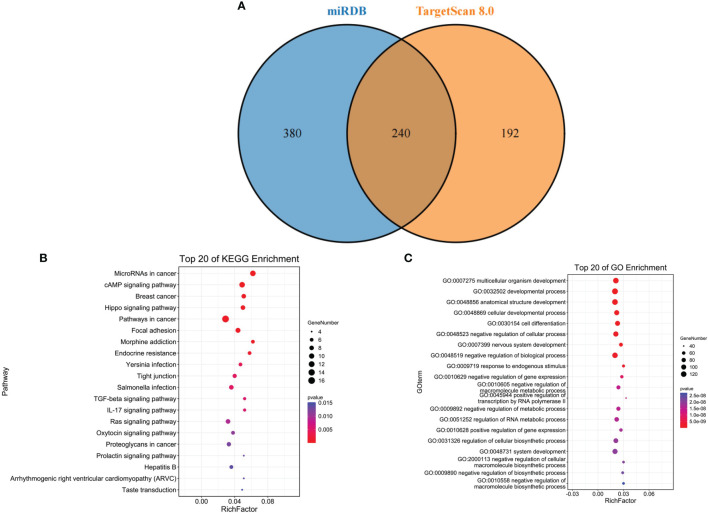
Target gene prediction of miR-139-5p. **(A)** miR-139-5p in the miRDB database with target gene overlap genes in the TargetScan 8.0 database; **(B)** Overlap gene KEGG pathway enrichment analysis; **(C)** Overlap gene GO function enrichment analysis.

The target genes of miR-139-5p were predicted by miRDB and TargetScan 8.0, respectively. Six hundred and twenty target genes were obtained in miRDB, 432 target genes in TargetScan 8.0, and 240 target genes were obtained after overlapping (**Figure 4A**); the KEGG and GO functional enrichment analyses were performed on the 240 target genes. The results showed that the 240 target genes were mainly enriched in cancer-related pathways (**Figure 4B**) and multicellular biological functions (**Figure 4C**).

### Expression and Prognosis of predicted Target Genes and Differentially Upregulated Genes

**Figure 5:**
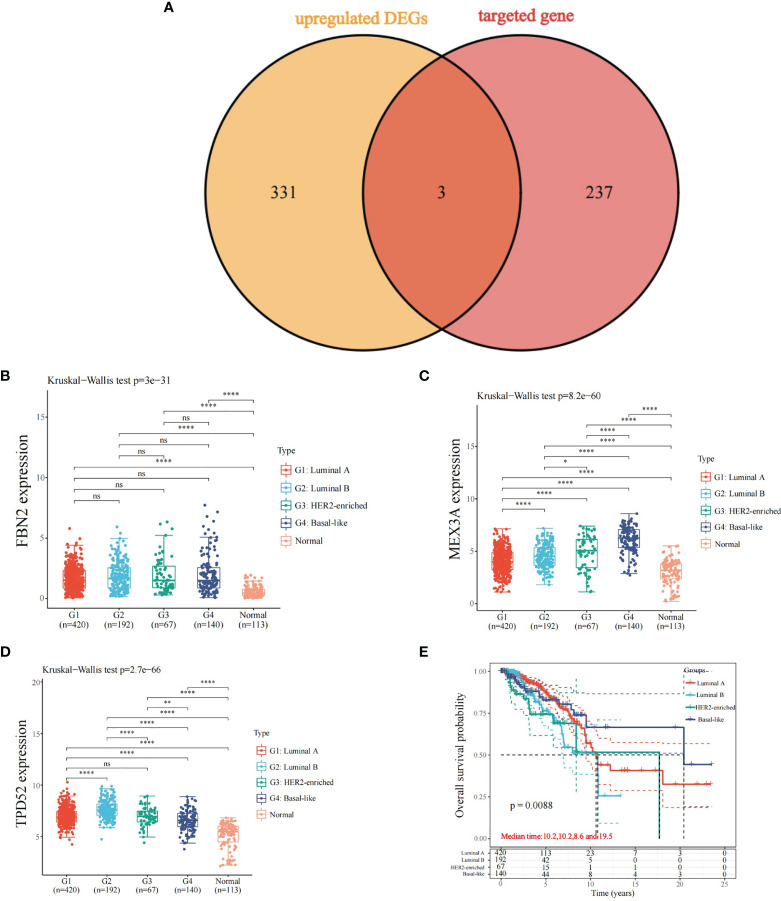
Expression and prognosis of predicted target genes and differentially up-regulated genes. **(A)** Overlapping genes of predicted target genes and differentially up-regulated genes; **(B)** Expression of FBN2 in different types of breast cancer; **(C)** Expression of MEX3A in different types of breast cancer; **(D)** Expression of TPD52 in different types of breast cancer; **(E)** Overall survival time of different types of breast cancer; *P < 0.05, ****P < 0.0001. ns, no significance.

A total of three key genes were obtained after overlapping the predicted target genes with the differentially upregulated genes in the four types of breast cancer: FBN2, MEX3A, and TPD52 (**Figure 5A**). Their expression and prognosis in the four types of breast cancer were observed separately, and the results showed that FBN2, MEX3A, and TPD52 were more expressed in luminal A, HER2, and TPD52 than in normal samples. The results showed that FBN2, MEX3A, and TPD52 were highly expressed in luminal A, luminal B, HER2-enriched, and basal-like breast cancers (**Figures 5B**–**D**) compared with normal samples. Besides, the overall survival time of different types of breast cancer were showed significant differences (**Figure 5E**).

### Functional Annotation of FBN2, MEX3A, and TPD52

**Figure 6:**
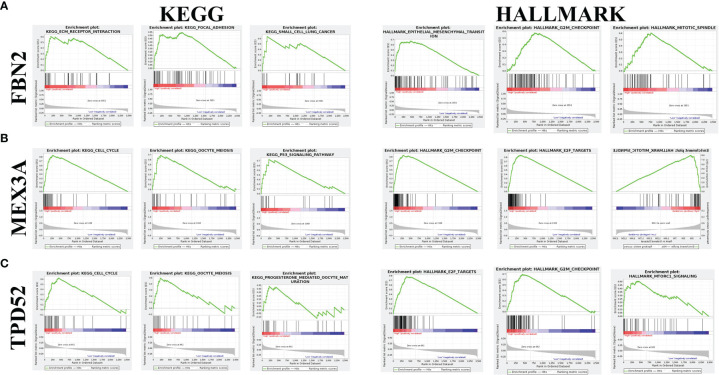
Enrichment analysis of FBN2, MEX3A, and TPD52 in KEGG and hallmark datasets. **(A)** Top three most relevant pathways of FBN2 in KEGG and hallmark databases; **(B)** Top three most relevant pathways of MEX3A in KEGG and hallmark databases; **(C)** Top three most relevant pathways of TPD52 in KEGG and hallmark databases.

As shown in **Figure 6**, the three KEGG pathways and hallmark pathways most significantly associated with high expression of FBN2, MEX3A, and TPD52 are given. Among them, the high expression of FBN2 was mainly enriched in extracellular matrix (ECM) receptor interaction and epithelial-mesenchymal transition (EMT) pathway (**Figure 6A**); the high expression of MEX3A was mainly enriched in cell cycle and G2M checkpoint (**Figure 6B**); and the high expression of TPD52 was mainly enriched in cell cycle and E2F targets (**Figure 6C**).

### Exosome Identification of BMSCs

**Figure 7:**
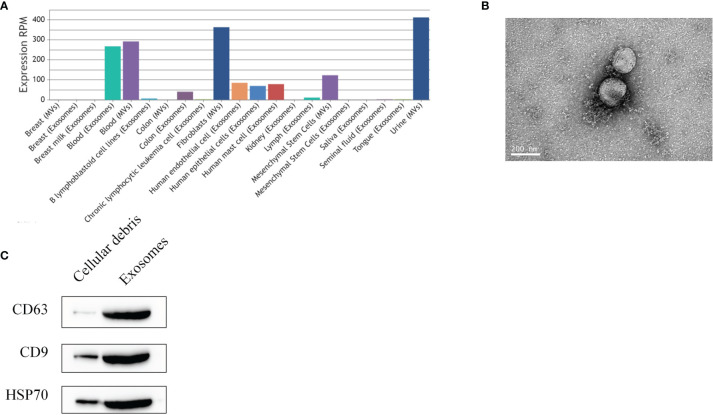
Identification of exosomes. **(A)** hsa-miR-139-5p expression of extracellular vesicles; **(B)** Transmission electron microscopy observation of exosomes; **(C)** Western blot detection of exosomal surface marker proteins of BMSCs.

By using the exosome miRNA database (http://bioinfo.life.hust.edu.cn/EVmiRNA#!/), the search revealed that miR-139-5p can be specifically expressed in MSCs (**Figure 7A**). Transmission electron microscopy showed that the exosomes were vesicle-shaped with a diameter of 100–200 nm (**Figure 7B**). The western blot showed that the exosomes could express specific proteins such as CD9, CD63, and HSP70 (**Figure 7C**).

### Expression of miR-139-5p and Its Target Genes in Cells and Effect on Proliferation

**Figure 8:**
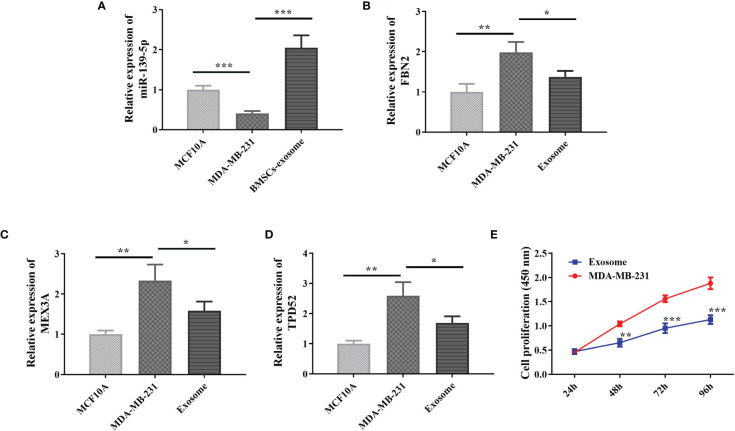
Expression of miR-139-5p and its target genes in cells and effect on proliferation. **(A)** Expression of miR-139-5p in cells; **(B)** Expression of FBN2 in cells; **(C)** Expression of MEX3A in cells; **(D)** Expression of TPD52 in cells; **(E)** Effect of BMSCs exosomes on cell proliferation, compared with control, *P < 0.05, **P < 0.01, ***P < 0.001).

In order to further analyze the effect of miR-139-5p on MDAMB-231 cells and its target genes, we examined the expression of miR-139-5p in cells and exocrine and its effect on the proliferation of MDA-MB-231 cells. The qPCR results showed (**Figures 8A–D**) that miR-139-5p expression was decreased in the MDA-MB-231 cells, and FBN2, MEX3A, and TPD52 expression was increased compared with the MCF-10A cells, and the differences were all statistically significant (P < 0.05). Further detection of miR-139-5p expression in exosomes showed that miR-139-5p expression was significantly higher in BMSCs exosomes than in MDA-MB-231 cells. In addition, FBN2,MEX3A, and TPD52 expression were significantly lower in MDA-MB-231 cells co-cultured with exosomes compared with MDA-MB-231 cells (P < 0.05). The CCK-8 results showed (**Figure 8E**) that after 48 h co-culture of exosomes with MDAMB-231 cells, compared with MDA-MB-231 cells, cell viability was significantly reduced in the exosome group compared with the MDA-MB-231 cells (P < 0.05).

## Discussion

Breast cancer remains one of the leading causes of death in women worldwide. Attributed to clinical, pathological, and biological factors, it is defined as a heterogeneous disease and, as such, presents differently in different populations, and treatment modalities should be individualized (22). The St. Gallen Consensus has led the development of personalized treatment for clinical and biological subtypes of breast cancer over the years. The Consensus can also be used to make informed adjuvant treatment decisions (23). However, the prevalence of molecular subtypes of breast cancer has not been extensively studied in developing countries. Over the past 20 years, scientists have studied the role of microRNAs in cancer development. An attractive advantage of microRNAs as cancer biomarkers is their stability in circulating fluid compared with other RNA species, allowing for noninvasive detection and tumor surveillance (24, 25). Numerous studies of differentially expressed genes and mRNAs have shown that miR-139-5p is one of the most important miRNAs in tumorigenesis, and an additional advantage is the ability of a single miRNA to simultaneously regulate many downstream signaling pathways (26).

In this study, we first analyzed the expression of miR-139-5p in different molecular subtypes of breast cancer and found that miR-139-5p was lowly expressed in luminal A, luminal B, HER2-enriched, and basal-like breast cancers, and the expression was significantly different in different molecular subtypes. Some studies have confirmed that miR-139-5p is lowly expressed in breast cancers (27), and the results of the present study also confirmed this finding and found that expression differences existed in different molecular subtypes. Subsequently, we identified three key genes, FBN2, MEX3A, and TPD52, by predicting miR-139-5p target genes and overlaying them with differentially expressed genes in different molecular subtypes, and selected differentially upregulated genes. Fibrillin-2 (FBN2), first expressed at the site of contact between epithelial and mesenchymal cells, is an extracellular calcium-binding microfibril involved in multiple biological pathways, including bone mineralization, osteoblast maturation, and calcium-binding (28). It has been identified as a diagnostic biomarker for smooth muscle sarcoma and rhabdomyosarcoma (29, 30). However, studies on FBN2 have focused on its methylation, and aberrant methylation of FBN2 has been found in breast cancer, non-small cell lung cancer, and esophageal squamous cell carcinoma (31–33), and the promoter region of the FBN2 gene has been repeatedly reported to be hypermethylated in several types of cancer. However, methylation may actually lead to downregulation of FBN2 in primary tumors (34). In contrast, FBN2 was found to be upregulated in different molecular subtypes of breast cancer in the present study, and this result, although different from the findings of previous studies, gives rise to the idea to study the role of FBN2 in a deeper way. MEX3A belongs to a family of RNA-binding proteins, consisting of four members (MEX3A-D). Many studies have pointed out that MEX3A is involved in mRNA regulation and influences the development of various diseases, especially human cancers, such as gastric cancer, bladder cancer, colorectal cancer, liver cancer, and glioblastoma. Jiang et al. (35) found that MEX3 was highly expressed in triple-negative breast cancer and promoted the proliferation and migration of triple-negative breast cancer through a PI3K/AKT signaling pathway. Shi et al. (36) found that MEX3A promotes the development of breast cancer by regulating PIK3CA. In the present study, not only was MEX3A highly expressed in different molecular subtypes of breast cancer, but also the expression was significantly different among the four different molecular subtypes, indicating the specificity of MEX3A expression. Therefore, we suggest that MEX3A may be a potential target for personalized treatment for different molecular subtypes of breast cancer. The tumor protein D52 (TPD52) is amplified from the human chromosome 8q21 amplification region to an oncogene and is highly expressed in many cancers, such as ovarian cancer and prostate cancer (37, 38). In addition, TPD52 is also overexpressed in breast cancer (39). There is increasing evidence that TPD52 is involved in cell transformation, proliferation, apoptosis, and metastasis (40, 41). Zhang et al. (39) found that TPD52 expression was significantly increased in breast cancer tissues and cells, and miR-449 deletion promoted proliferation and metastasis of breast cancer cells by regulating TPD52. In contrast, in the present study, whether TPD52, a target gene of miR-139-5p, plays a role in the proliferation and metastasis of breast cancer cells needs to be further investigated.

To further demonstrate the regulatory role of miR-139-5p in breast cancer, we found that miR-139-5p was expressed in the extracellular bodies of mesenchymal stem cells. In addition, bone MSCs have been shown to contribute to tumorigenic processes, including proliferation, metastasis, and drug resistance in a variety of cancers, mainly through the secretion of paracrine factors or cell-cell interactions (42, 43). Therefore, we used human BMSCs to isolate and obtain their exosomes to overexpress miR-139-5p as a transporter of miR-139-5p into breast cancer cells. The isolated products were confirmed to be exosomes and expressed miR-139-5p. Subsequently, they were co-cultured with MDA-MB-231 cells and found to inhibit the proliferation ability of MDA-MB-231 cells. In previous studies, miR-139-5p has been identified as a tumor suppressor in breast (44), colorectal (45) and esophageal squamous cell carcinoma (17), where it has been shown to inhibit migration, invasion, and metastasis. In this study, we found that miR-139-5p not only can be a potential biomarker in breast cancer, but also in different molecular subtypes of breast cancer. miR-139-5p may be a good candidate for further research in advanced preclinical studies as a therapeutic target, not only for breast cancer, but also for solid tumors.

In summary, this study revealed by comprehensive biological information analysis that miR-139-5p was lowly expressed in various kinds of breast cancer, and miR-139-5p was highly expressed in exosomes rooted in BMSCs. Bone marrow mesenchymal stem cells-derived exosomal miR-139-5p inhibited the proliferation of MDA-MB-231 cells, laying an experimental foundation for the clinical application of exosomal miR-139-5p gene in breast cancer treatment. In addition, the key target genes of miR-139-5p, FBN2, MEX3A, and TPD52 also provide potential targets for personalized treatment of different molecular subtypes of breast cancer.

## Data Availability Statement

The original contributions presented in the study are included in the article/supplementary material. Further inquiries can be directed to the corresponding author.

## Authors Contribution

All authors listed have made a substantial, direct, and intellectual contribution to the work and approved it for publication.

## Funding

This work was supported by Ningbo Medical Science and Technology Program Project (2018A29), Ningbo Natural Science Foundation Project (2019A610302), Zhejiang Province Medical and Health Clinical Research Application Project (2022KY1173).

## Conflict of Interest

The authors declare that the research was conducted in the absence of any commercial or financial relationships that could be construed as a potential conflict of interest.

## Publisher’s Note

All claims expressed in this article are solely those of the authors and do not necessarily represent those of their affiliated organizations, or those of the publisher, the editors and the reviewers. Any product that may be evaluated in this article, or claim that may be made by its manufacturer, is not guaranteed or endorsed by the publisher.
